# Perceptions of asthma control in the United Kingdom: a cross-sectional study comparing patient and healthcare professionals’ perceptions of asthma control with validated ACT scores

**DOI:** 10.1038/s41533-017-0050-x

**Published:** 2017-08-11

**Authors:** Andrew Menzies-Gow, Gavin Chiu

**Affiliations:** 1 Royal Brompton Hospital, London, SW3 6NP UK; 2 Boehringer Ingelheim UK, Bracknell, RG12 8YS UK

## Abstract

Perceptions of asthma control often vary between patients and physicians. This cross-sectional survey provided UK-specific data on actual and perceived asthma control in patients (18–75 years) attending routine asthma reviews in primary, secondary and tertiary settings. Differences between healthcare professionals’ (HCP) and patients’ perceptions of asthma control were evaluated via an online questionnaire and compared to a control—the validated asthma control test (ACT)—which patients completed. Treated patients (at least a short acting ß-agonist) with a documented diagnosis of asthma were enroled and consented within a month of their last appointment. Patients were grouped according to the British Thoracic Society (BTS)/Scottish Intercollegiate Guidelines Network (SIGN) 2014 treatment guidelines (BTS/SIGN steps 1–5). A total of 260 patients were screened: 234 were eligible for enrolment: 33, 52, 50, 49 and 50 patients in steps 1–5, respectively. Seventy per cent (164) were women. The percentage of patients aged 45–64 years was 47.4%. HCPs classed 70% (164) as non-smokers. 84.2% of patients and 73.9% of HCPs perceived that asthma was controlled but ACT results suggest that asthma was only controlled in 54.7% of patients (ACT score ≥20). Patients in steps 4 and 5 had the highest levels of uncontrolled asthma. Correct agreement between ACT score with perceptions of controlled or uncontrolled asthma occurred in 67.9% of patients and 68.8% of HCPs; the poorest levels of agreement occurred in patients in steps 4 and 5. Uncontrolled asthma is common in UK patients. High proportions of patients and HCPs have incorrect perceptions of asthma control, especially in relation to patients with asthma in steps 4 and 5.

## Asthma: Misperceptions of control abound among UK patients and doctors

Asthma patients and their doctors often misperceive whether the disease is under control, according to a British study. Andrew Menzies-Gow from Royal Brompton Hospital in London and Gavin Chiu from Boehringer Ingelheim UK in Bracknell used an online questionnaire to assess perceptions of asthma control among 234 patients and their healthcare providers. All participants had at least intermittent asthma that required occasional treatment; many had more severe disease. The researchers found that 84% of patients and 74% of doctors thought the asthma was well controlled, but results of the Asthma Control Test indicated only 55% of patients objectively achieved disease control. Correct agreement between the validated test and more subjective perceptions occurred in only 68 % of patients and 69% of doctors. Addressing this mismatch could go a long way to improving asthma control among British patients.

## Introduction

Despite the existence of evidence-based guidelines and availability of effective medications, asthma is still a major health problem in the United Kingdom.^1, 2^ UK patients experience a high prevalence of poor asthma control, which results in the need for both controller and rescue medication; an increased likelihood of exacerbations; and high rates of emergency healthcare utilisation, hospitalisation and death.^2–4^ Data from a European study, conducted in 2006, demonstrated that 10% of the UK population had been diagnosed with asthma (*n* = 4.67 million), and 42.7% of these patients (1.99 million) had not well-controlled (NWC) asthma.^4^ The 2014 National Review of Asthma Deaths reported that the majority of people who died from asthma in the United Kingdom between February 2012 and January 2013 had not been receiving specialist care in the 12 months prior to death (112/195, 57%).^1^ Nearly half of the patients had a history of having been admitted to the hospital because of asthma (90/190, 47%). Both physician- and patient-related factors were identified in a substantial proportion of patients who died. The authors concluded that many patients who were treated as having mild or moderate asthma had poorly controlled, under-treated asthma rather than mild or moderate disease. They recommended that an assessment of recent asthma control should be undertaken at every asthma review; and appropriate interventions should be implemented for patients with NWC asthma. It has been suggested that it is not asthma per se that causes the burden of illness but uncontrolled asthma.^4^

A number of factors have been identified as contributing to poor levels of asthma control in the United Kingdom: inadequate provision and updating of personal asthma action plans; under-treatment of mild–moderate asthma; lack of medical assessment of factors that trigger or exacerbate asthma; poor inhaler technique; inadequate adherence to medication; patients’ lack of knowledge about the causes and triggers of asthma; and a poor perception of what constitutes well-controlled asthma among both HCPs and patients.^1, 5^ Given that the British Thoracic Society (BTS)/Scottish Intercollegiate Guidelines Network (SIGN) 2014 treatment guidelines^5^ are based on patient-reported symptoms as well as HCP-identified symptoms, appropriate treatment may not be prescribed if a patient does not understand what NWC asthma is, and does not report his/her symptoms to the HCP. Patients who do not report symptoms run the risk of under-treatment and of NWC asthma with its consequent morbidities.

The asthma control test (ACT) is a self-administered, online, validated questionnaire that enables a patient to evaluate his/her level of asthma control over the preceding 4 weeks (http://www.asthmacontroltest.com).^6, 7^ ACT scores ≥20 identifies well-controlled asthma and scores <20 represent not well-controlled asthma.

The rationale for conducting this study was to provide UK-specific data on levels of asthma control in a cross-section of the UK population. This will help us to gain a better understanding of how common misperception of asthma control is and the differences in perceptions that may occur between HCPs and patients.

The objectives of the study were:

1. To determine rates of poor asthma control in a cohort of patients attending routine asthma reviews in the primary, secondary and tertiary UK care settings using ACT.
2. To assess the rates of uncontrolled asthma in patients with asthma in steps 1–5, as described in the BTS/SIGN 2014 guidelines.
3. To assess concordance between patients’ perception of their asthma control and their ACT results.
4. To assess concordance between the HCPs’ perception of the patient’s asthma control and the patients’ ACT results.
5. To compare patients’ and HCPs’ perceptions of asthma control in relation to the patients’ ACT scores.

## Results

### Baseline data

The recruitment target was 320 but this was not achieved during the study period. It proved particularly difficult to recruit a sufficient number of patients in step 1 . A total of 260 patients were screened: 234 were eligible for the study: 33, 52, 50, 49 and 50 patients in steps 1–5, respectively (full analysis set (FAS)) (Table 1). The per protocol set (PPS) was very similar to the FAS since it comprised 97% (*n* = 227) of the FAS: 33, 51, 48, 48 and 47 patients in steps 1–5, respectively.

**Table 1:** Baseline demographics and disease characteristics

	BTS/SIGN step (2014 guidelines)					
	Step 1	Step 2	Step 3	Step 4	Step 5	Overall
Number of patients (*N*, %)	33 (100.0)	52 (100.0)	50 (100.0)	49 (100.0)	50 (100)	234 (100.0)
Sex (*N*, %)						
Male	11 (33.3)	18 (34.6)	16 (32.0)	13 (26.5)	12 (24.0)	70 (29.9)
Female	22 (66.7)	34 (65.4)	34 (68.0)	36 (73.5)	38 (76.0)	164 (70.1)
Age group (*N*, %)						
18–24	5 (15.2)	4 (7.7)	3 (6.0)	2 (4.1)	0 (0.0)	14 (6.0)
25–34	7 (21.2)	8 (15.4)	4 (8.0)	3 (6.1)	10 (20.0)	32 (13.7)
35–44	8 (24.2)	5 (9.6)	8 (16.0)	9 (18.4)	5 (10.0)	35 (15.0)
45–54	3 (9.1)	11 (21.2)	7 (14.0)	20 (40.8)	17 (34.0)	58 (24.8)
55–64	5 (15.2)	14 (26.9)	12 (24.0)	8 (16.3)	14 (28.0)	53 (22.6)
65–75	5 (15.2)	10 (19.2)	16 (32.0)	7 (14.3)	4 (8.0)	42 (17.9)
Asthma diagnosis, years (*N*, %)						
0–5	5 (15.2)	10 (19.2)	7 (14.0)	8 (16.3)	6 (12.0)	36 (15.4)
6–10	3 (9.1)	8 (15.4)	7 (14.0)	6 (12.2)	4 (8.0)	28 (12.0)
11–20	9 (27.3)	14 (26.9)	14 (28.0)	8 (16.3)	9 (18.0)	54 (23.1)
21–30	7 (21.2)	9 (17.3)	6 (12.0)	10 (20.4)	9 (18.0)	41 (17.5)
>30	9 (27.3)	11 (21.2)	16 (32.0)	17 (34.7)	22 (44.0)	75 (32.1)
Smoking status (*N*, %)						
Non-smoker	20 (60.6)	37 (71.2)	33 (66.0)	41 (83.7)	33 (66.0)	164 (70.1)
Ex-smoker	11 (33.3)	12 (23.1)	15 (23.1)	8 (16.3)	17 (34.0)	63 (26.9)
Current smoker	2 (6.1)	3 (5.8)	2 (4.0)	0 (0.0)	0 (0.0)	7 (3.0)
Hospital admission (*N*, %)						
No	33 (100.0)	52 (100.0)	49 (98.0)	34 (69.4)	38 (76.0)	206 (88.0)
Yes	0 (0.0)	0 (0.0)	1 (2.0)	15 (30.6)	12 (24.0)	28 (12)
Medication change (*N*, %)						
Yes	28 (84.8)	42 (80.8)	32 (64.0)	15 (30.6)	25 (50.0)	142 (60.7)
No	5 (15.2)	10 (19.2)	18 (36.0)	34 (69.4)	25 (50.0)	92 (39.3)
Exacerbations^a^ (*N*, %)						
0	28 (84.8)	31 (59.6)	29 (58.0)	6 (12.2)	3 (6.0)	97 (41.5)
1	4 (12.1)	16 (30.8)	8 (16.0)	7 (14.3)	12 (24.0)	47 (20.1)
2	1 (3.0)	3 (5.8)	8 (16.0)	13 (26.5)	8 (16.0)	33 (14.1)
3	0 (0.0)	1 (1.9)	2 (4.0)	7 (14.3)	7 (14.0)	17 (7.3)
4	0 (0.0)	1 (1.9)	1 (2.0)	4 (8.2)	10 (20.0)	16 (6.8)
5	0 (0.0)	0 (0.0)	0 (0.0)	2 (4.1)	3 (6.0)	5 (2.1)
>5	0 (0.0)	0 (0.0)	2 (4.0)	10 (20.4)	7 (14.0)	19 (8.1)

^a^ An exacerbation was defined as 3 days of oral steroids or doubling of usual maintenance oral steroid dose.

Females accounted for 70% (*n* = 164) of the study population (Table 1). Nearly half of the patients (47.4%, *n* = 111) were aged 45–64 years. One-third of patients (32.1%, *n* = 75) had been diagnosed with asthma >30 years before they completed the questionnaire. Medications taken at baseline are summarised in Table 2. Documenting the patient’s medication(s) allowed the patient’s treatment step (1–5) to be identified.^5^ Several patients were assigned to the inappropriate step based on the medications they were taking: seven patients in steps 1–2 were taking a long acting/inhaled corticosteroid; and 32 patients in step 5 were not taking oral corticosteroids (Table 2).

**Table 2:** Medications taken at baseline

	BTS/SIGN Step (2014 guidelines)					
	Step 1	Step 2	Step 3	Step 4	Step 5	Overall
Long acting/inhaled corticosteroid (*N*, %)						
No	32 (97.0)	46 (88.5)	10 (20.0)	5 (10.2)	3 (6.0)	96 (41.0)
Yes	1 (3.0)	6 (11.5)	40 (80.0)	44 (89.8)	47 (94.0)	21 (9.0)
Montelukast (*N*, %)						
No	33 (100.0)	52 (100.0)	44 (88.0)	15 (30.6)	31 (62.0)	175 (74.8)
Yes	0 (0.0)	0 (0.0)	6 (12.0)	34 (69.4)	19 (38.0)	59 (25.2)
Aminophylline (*N*, %)						
No	33 (100.0)	52 (100.0)	50 (100.0)	40 (81.6)	34 (68.0)	209 (89.3)
Yes	0 (0.0)	0 (0.0)	0 (0.0)	9 (18.4)	16 (32.0)	25 (10.7)
Omalizumab (*N*, %)						
No	33 (100.0)	52 (100.0)	50 (100.0)	37 (75.5)	11 (22.0)	183 (78.2)
Yes	0 (0.0)	0 (0.0)	0 (0.0)	12 (24.5)	39 (78.0)	51 (21.8)
Oral corticosteroids (*N*, %)						
No	33 (100.0)	52 (100.0)	50 (100.0)	44 (89.8)	32 (64.0)	211 (90.2)
Yes	0 (0.0)	0 (0.0)	0 (0.0)	5 (10.2)	18 (36.0)	23 (9.8)
Oral ß2-agonists						
No	33 (100.0)	52 (100.0)	50 (100.0)	49 (100.0)	49 (98.0)	233 (99.6)
Yes	0 (0.0)	0 (0.0)	0 (0.0)	0 (0.0)	1 (2.0)	1 (0.4)

Data on comorbidities and other patient characteristics, e.g., socio-economic status, were not collected because this was an exploratory study conducted in a ‘real-world’ setting.

HCPs and patients were independently asked about the patient’s reported smoking history. The majority of patients were non-smokers (*n* = 164/234, 70.1%). Among the 63 ex-smokers, the median number of pack years was 4, with a range of 0.2–30. Only seven patients were current smokers; the median pack years for this group was 5.25 (range, 2.0–9.0).

### ACT scores versus perception of asthma control

**Fig. 1:**
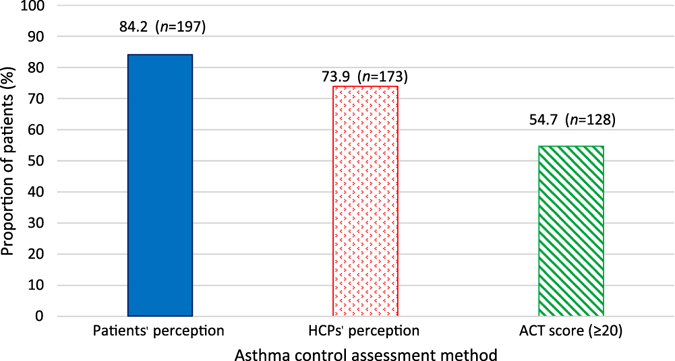
Total population reporting controlled asthma (*n* = 234). *ACT* asthma control test (ACT score ≥20 represents good asthma control), *HCP* healthcare professional. Percentages are based on the number of patients in the full analysis set

The ACT results suggest that asthma was controlled in 54.7% of patients (ACT score ≥20), with levels of uncontrolled asthma highest in patients in steps 4 and 5. 84.2% of patients and 73.9% of their HCPs perceived that their asthma was controlled (Fig. 1).

**Fig. 2:**
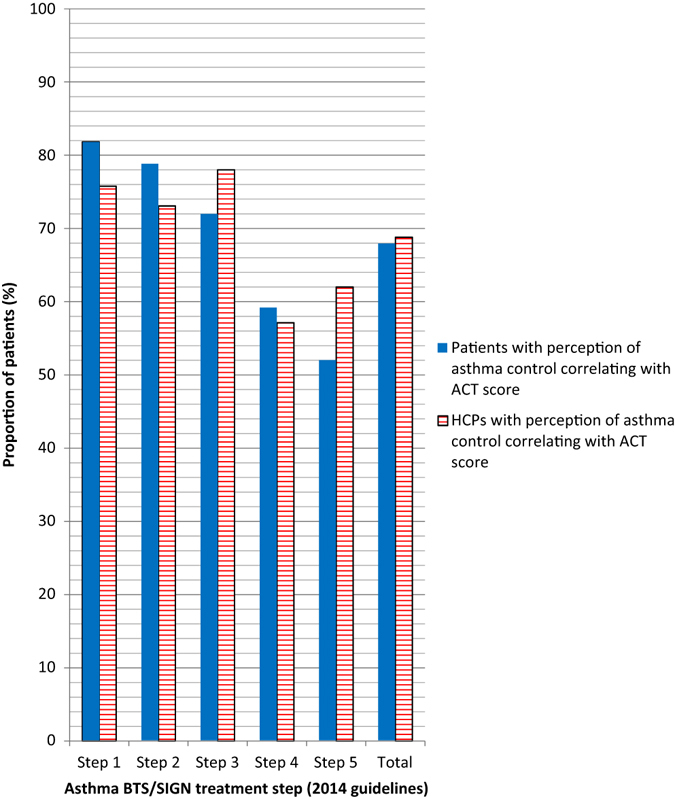
Perception of asthma control correlated with ACT score: patient vs. HCPs (*n* = 234). *ACT* asthma control test, *BTS/SIGN* British Thoracic Society/Scottish Intercollegiate Guidelines Network, *HCP* healthcare professional. Percentages are based on the number of patients in the full analysis set. BTS step 1 = Mild intermittent asthma, step 2 = Regular preventer therapy, step 3 = Initial add-on therapy, step 4 = Persistent poor control, step 5 = continuous or frequent use of oral steroids (2014 guidelines)

**Fig. 3:**
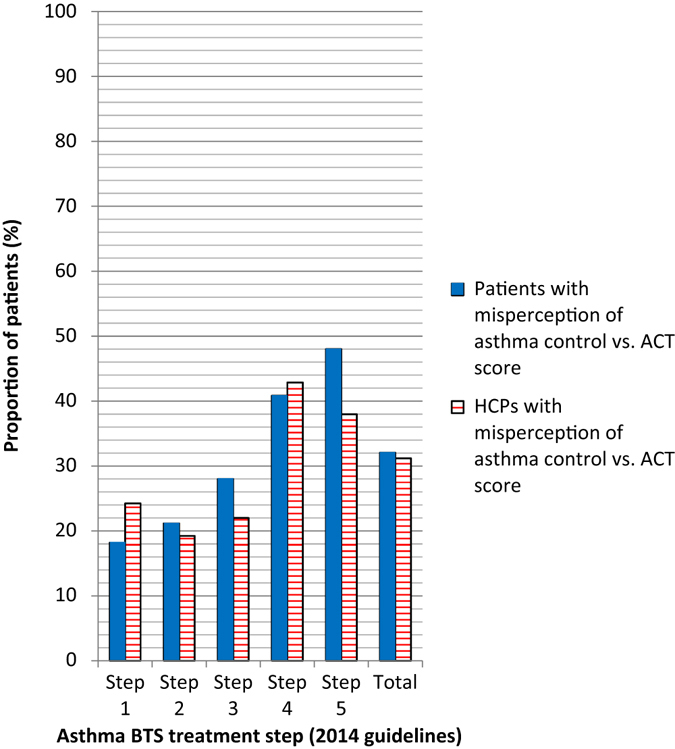
Perception of asthma control did not correlate with the ACT score: patients vs. HCPs. *ACT* asthma control test, *BTS/SIGN* British Thoracic Society/Scottish Intercollegiate Guidelines Network, *HCP* healthcare professional. Percentages are based on the number of patients in the full analysis set. BTS step 1 = Mild intermittent asthma, step 2 = Regular preventer therapy, step 3 = Initial add-on therapy, step 4 = Persistent poor control, step 5 = continuous or frequent use of oral steroids (2014 guidelines)

**Fig. 4:**
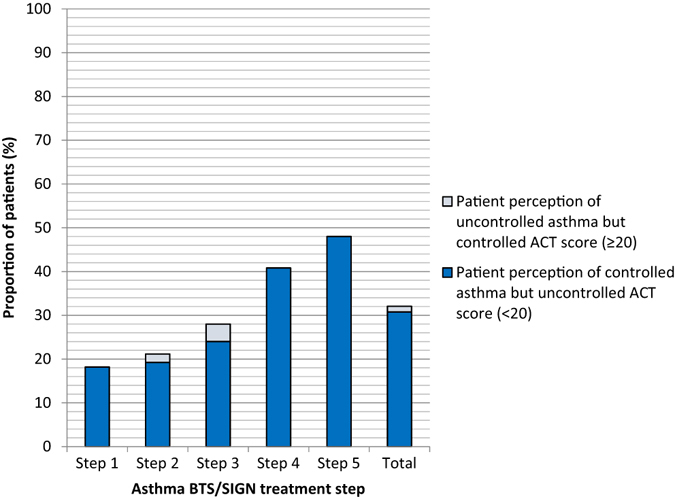
Patient perception of asthma control compared to their ACT score

Correct agreement between ACT score with perception of controlled or uncontrolled asthma occurred in 67.9% of patients and 68.8% of HCPs; the poorest levels of agreement occurred in patients in steps 4 and 5 (Fig. 2). Of the 128 patients who had ACT scores ≥20, 97.7% (*n* = 125) believed that their asthma was controlled and 114/128 (89.1%) HCP assessments were in concordance with the ACT scores. Only 34/106 (32.1%) patients with ACT scores <20 and 47/106 (44.3%) of their HCPs thought that the asthma was uncontrolled (Fig. 3). The majority of these 106 patients (59/106, 55.7%) and their HCPs (59/106, 55.7%) believed that their asthma was controlled even though the objective measurement indicated otherwise. Patients in steps 1–3 were more likely have an accurate perception of their level of asthma control than those in steps 4 and 5 (Figs. 3 and 4). A minority of patients perceived that they had uncontrolled asthma when their ACT score indicated otherwise (ACT score ≥20) (Fig. 4). Nearly one-third of patients (30.8%) believed they had controlled asthma when their ACT score was <20, indicating uncontrolled asthma; this proportion was higher in steps 4 (41%) and 5 (48%) patients. Similarly, HCPs managing patients in steps 4 and 5 were more likely to think that asthma control had been achieved, even though the ACT score indicated that it had not been, than HCPs treating patients in steps 1–3.

## Discussion

### Main findings

Relationships between patient and HCP perceptions of asthma control and objective measurement of asthma control (via the ACT score) were explored in the study reported in this paper. Both patients and HCPs considered that asthma was better controlled than the ACT scores indicated. It is noteworthy that patients were more likely to overestimate the level of asthma control achieved than HCPs. A small proportion of patients thought that their asthma was not controlled, even though their ACT results indicated good levels of control. It is possible that these patients were being over-treated because of a misperception about their level of asthma control, and were, therefore, at risk of the adverse effects of the excessive medication.

Although HCPs were slightly better than patients at classifying poor asthma control correctly, they did not identify 100% of patients who needed further intervention to achieve good control. In particular, they overestimated levels of asthma control in patients in steps 4 and 5, who are at the most risk of exacerbations and adverse outcomes. This could be a chance finding because the study lacked the power to make comparisons between the different steps. Differences in the age distribution of patients in each step may also have affected the comparisons. Alternatively, overestimation of control may be due to HCPs’ tendency to assess asthma control on the basis of patients’ self-reports of symptoms and/or their conviction that the current medication was effective. These factors should be explored further so that HCP assessment of levels of asthma control can be improved.

### Interpretation of findings in relation to previously published work

These data are in agreement with the results of similar studies that have identified high levels of uncontrolled asthma in UK patients, especially in patients in steps 4 and 5 (2014 guidelines).^2, 4, 8^ Data from the 2006 European National Health and Wellness Survey indicated that 44.8% (1.88 million) of the 4.67 million UK patients who were being treated for asthma had NWC asthma.^4^ When compared with ACT scores, half of the European patients studied (50.4%, 7.09 million across Europe) did not have well-controlled asthma but 64.7% of patients considered that their asthma was controlled. The REALISE investigators also concluded that asthma control in Europe is poor: >80% of the 8000 patients surveyed in 2012 considered their asthma to be controlled, even though objective measurements suggested 45% had NWC asthma and 34.8% had partially controlled asthma.^2^ Nearly one-fifth (19.5%) of patients who did not regard their asthma as serious had visited the emergency department in the previous year for asthma-related reasons. The United Kingdom has one of the highest asthma-related death rates in Europe: more than 20 people die from the disease each week.^9^

The REALISE study identified a high level of oral steroid use, emergency department visits and hospitalisations in the 12 months prior to the survey, even in patients whose asthma was controlled at the time of the survey.^2^ An analysis of UK 2010–2011 data showed that 21.5% of NWC patients in a sample of 701 UK patients with asthma reported visits to the Accident and Emergency department compared to 14.1% of patients with well-controlled asthma.^10^ In addition, patients with NWC asthma were more likely to visit their doctors (both GPs and specialists) or be hospitalised than patients with well-controlled asthma. Poor asthma control was associated with several negative outcomes, such as diminished health-related quality of life, high use of healthcare resources, and work and activity impairment.^10^ A study published in 2013 estimated the total direct cost of treating asthma in the United Kingdom as over £758 million.^11^ Although only ~2.7% of the study population had uncontrolled asthma and multiple exacerbations, their care accounted for nearly £53 million (~7% of care costs).^11^

### Strengths and limitations of this study

A major strength of this study is that it was conducted in clinical practice rather than a study setting. Patients were attending routine asthma reviews in primary, secondary and tertiary clinics. They represent a ‘real-world’ sample of patients across the clinical spectrum of asthma (steps 1–5 of the BTS/SIGN 2014 guidelines) rather than a pre-selected group of patients (e.g., in tertiary care or only patients in step 1) who would be eligible for recruitment into a trial. Hence, these results can be applied to a wide range of patients. The data can be used to educate both patients and HCPs about the need to critically evaluate the effectiveness of therapy in order to prevent under- and over-treatment.

The CONSORT statement does not apply to this study since it was not a randomised trial. The interpretation of data from this study should take into account that it was an observational study of a non-randomised sample of patients. The generalisation of the results to the target population of patients with asthma has not been established. Assessments of asthma control were limited to the previous 4 weeks and may have been subject to poor or incomplete recall.

The sample size (*n* = 234) was too small to provide the power for detailed statistical comparisons between patients with different levels of asthma severity. The study design meant that it was not possible to perform sensitivity analyses to examine the possible impact of potential confounders such as comorbidities or age on the study outcomes. A larger, randomised study, with sufficient statistical power to determine differences between the various steps and that assessed patients using current BTS/SIGN guidelines (www.brit-thoracic.org.uk), could be conducted to define levels of asthma control in patients at different points along the disease spectrum. Monitoring adherence to medication would provide useful information about the impact of adherence on asthma control.

Although the ACT scoring system has been validated, its accuracy depends on patient-reported symptoms, and so there is an element of subjectivity in the assessment. Mechanical measurements of lung function provide more objective data than the ACT, but are more onerous to carry out, especially in primary and secondary settings. The ACT is a practical method of assessing levels of asthma control in a range of settings.

### Implications for future research, policy and practice

Although the GINA guidelines recommend a stepwise therapeutic approach, the reported high levels of uncontrolled asthma suggest under-use or inappropriate use of medication or under-prescription of therapies.^12^ HCP education and patient empowerment are both necessary to maximise asthma control.^4, 5, 12^ Completing the ACT in the clinic would provide data to support a HCP’s decision to prescribe appropriate medication in order to optimise asthma control and improve patient outcome.^13^ Access to online self-management strategies, including monitoring of control, treatment advice and online education, has been shown to improve asthma control in Dutch patients.^14^

The BTS/SIGN guidelines state that ‘the best predictor of future asthma attacks is current control’ and ‘the benefits of inflammation guided management are greater in patients with severe asthma, when asthma attacks can occur frequently and unpredictably’.^5^ Therefore, attaining and maintaining optimal control should be the main therapeutic goal when managing patients with asthma, especially patients in steps 4 and 5.^5^

## Conclusions

This study has identified a major barrier to achieving the BTS/SIGN goal of effective asthma therapy in the United Kingdom: a lack of agreement between objective and subjective assessments of asthma control by both patients and HCPs. Overcoming this barrier should improve asthma control in UK patients.

## Methods

This study was approved by the NRES Committee North East – York on 2 May 2014 (reference 14/NE/0137). Patient consent was obtained prior to the study commencement.

The study was carried out in compliance with the Declaration of Helsinki, in accordance with the Good Epidemiological Practice – IEA Guidelines for proper conduct of epidemiological research and relevant standard operating procedures. Standard medical care (prophylactic, diagnostic and therapeutic procedures) was the responsibility of the patient’s HCP.

**Fig. 5:**
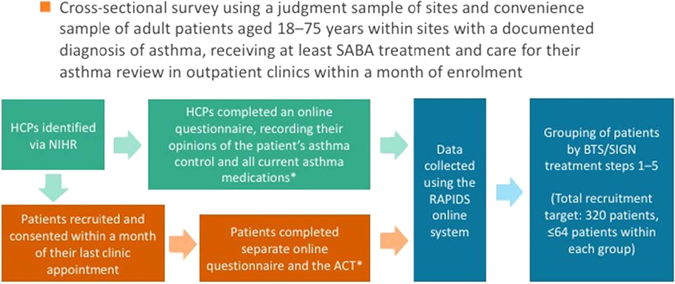
Study design. *ACT* asthma control test, *BTS/SIGN* British Thoracic Society/Scottish Intercollegiate Guidelines Network, *HCP* healthcare professional, *NIHR* the National institute for Healthcare Research, *SABA* short acting ß-agonist. *All assessment were conducted at a single time point, a cross-section in time. At this time, the patient completed the ACT, demographic data and perception of their asthma control in the period reflected in the ACT. Recruiting physicians completed information about the patient's medications and their perception of the patient's asthma control for the period covered by the ACT. No intervention or medication was assessed in this study. BTS step 1 = Mild intermittent asthma, step 2 = Regular preventer therapy, step 3 = Initial add-on therapy, step 4 = Persistent poor control, step 5 = continuous or frequent use of oral steroids

Adult patients were enroled in the study if they had a confirmed diagnosis of asthma; were being treated for the condition; and were attending routine asthma reviews in primary, secondary and tertiary settings (Fig. 5). Inclusion criteria were: age 18–75 years; documented diagnosis of asthma; treatment with at least a short acting β-agonist; and attendance for an asthma review in primary, secondary and tertiary care outpatient clinics within a month of enrolment. The aim was to enrol patients across the range of asthma severity: this was achieved by enroling subjects from a variety of different healthcare providers ranging from primary care to tertiary specialist severe asthma clinics. This was not a randomised study: a convenience sample of patients was recruited and consented by HCPs who were responsible for the patients’ follow-up within a month of their last clinic appointment. The investigators could use their discretion as to which subjects were approached and subsequently enroled in the study. Each HCP recorded the patient’s treatment history and his/her perception as to whether the asthma was well controlled or not via an online questionnaire. Each patient provided demographic data and his/her perception of asthma control via an online questionnaire, as well as completing the ACT. Patients answered the question: ‘In the last 4 weeks, do you feel that your asthma has been: controlled or uncontrolled’. Patients provided information about their smoking history (ex-smoker, current smoker, non-smoker). Smokers and ex-smokers were classified according to the number of years they had smoked and their smoking pack years (see statistical section for how pack years were calculated).

Patients were divided into five groups based on the BTS/SIGN treatment guidelines (steps 1–5) (2014 guidelines) (Fig. 5), based on their reported current treatment.^5^

Patients were excluded if they had a history of an asthma exacerbation in the last 4 weeks; a diagnosis of any chronic respiratory condition (such as chronic obstructive pulmonary disease or bronchiectasis); or a smoking history of >10 pack years.

### Data analysis

The study was not designed to achieve statistical power but to produce data for descriptive purposes. Patient data were analysed for the whole study population and for each BTS/SIGN step (2014 guidelines). The number of pack years was calculated for each patient who described him/herself as an ex- or current smoker, using the following equation:

$$\\ 	 {\rm{Pack}}\,{\rm{years = }}\left( {{\rm{patient}}\,{\rm{estimate}}\,{\rm{of}}\,{\rm{number}}\,{\rm{of}}\,{\rm{cigarettes}}\,{\rm{smoked}}\,{\rm{per}}\,{\rm{day/}}20} \right)\\ 	 *{\rm{number}}\,{\rm{of}}\,{\rm{years}}\,{\rm{smoked}}$$

No statistical comparisons were made among the BTS/SIGN groups. All analyses were conducted on the FAS, which included all patients who took part in the patient survey. A PPS excluded patients who had violated the study inclusion criteria.

There were no missing data since data were not submitted to the study database until all of the online fields of the questionnaires had been completed by the patient or HCP.

### Data availability

The data that support the findings of this study are available from the corresponding author on reasonable request.
